# Effect of cognitive-behavioral program on quality of life in men with post-prostatectomy incontinence: a randomized trial

**DOI:** 10.1590/1980-220X-REEUSP-2024-0187en

**Published:** 2024-10-28

**Authors:** Lívia Cristina de Resende Izidoro, Cissa Azevedo, M. Graça Pereira, Tania Couto Machado Chianca, Cristiane José Borges, Lizete Malagoni de Almeida Cavalcante Oliveira, Luciana Regina Ferreira da Mata

**Affiliations:** 1 Universidade Federal de Jataí, Jataí, GO, Brazil.; 2 Universidade Federal de São João del-Rei, Divinópolis, MG, Brazil.; 3 Universidade do Minho, Braga, Portugal.; 4 Universidade Federal de Minas Gerais, Escola de Enfermagem, Belo Horizonte, MG, Brazil.; 5 Universidade Federal de Goiás. Goiânia, GO, Brazil.

**Keywords:** Quality of Life, Urinary Incontinence, Prostatectomy, Cognitive Behavioral Therapy, Qualidade de Vida, Incontinência Urinária, Prostatectomia, Terapia Cognitivo-Comportamental, Calidad de Vida, Incontinencia Urinaria, Prostatectomia, Terapia Cognitivo-Conductual

## Abstract

**Objective:**

To explore the effects of a cognitive-behavioral program addressing urinary incontinence on the quality of life of men who have undergone radical prostatectomy.

**Method:**

Randomized controlled clinical trial with patients undergoing radical prostatectomy in an institution for cancer treatment in Brazil. The 34 participants were randomized into two groups: 17 in the control group who received the institution’s standard care and 17 in the intervention group who received the cognitive-behavioral program. Quality of life was assessed using the King’s Health Questionnaire and the International Consultation on Incontinence Questionnaire-Short Form.

**Results:**

Participants from intervention group showed better results regarding the reduction of the impact of urinary incontinence on quality of life (p ≤ 0.001), with emphasis on limitations in daily activities, general health perception, physical and social limitations, emotions, and sleep and mood.

**Conclusion:**

The cognitive-behavioral program was effective in reducing the impact of urinary incontinence on quality of life. This study contributes to clinical practice by providing an effective, low-cost, and easily applicable therapy. Brazilian Registry of Clinical Trials: RBR-3sstqg.

## RESUMO

**Objetivo:**

Explorar os efeitos de um programa cognitivo-comportamental que aborda a incontinência urinária na qualidade de vida de homens submetidos à prostatectomia radical.

**Método:**

Ensaio clínico controlado randomizado com pacientes submetidos à prostatectomia radical em instituição para tratamento de câncer no Brasil. Os 34 participantes foram randomizados em dois grupos: 17 no grupo controle, que recebeu o tratamento padrão da instituição, e 17 no grupo de intervenção, que recebeu o programa cognitivo-comportamental. A qualidade de vida foi avaliada usando o King’s Health Questionnaire e o International Consultation on Incontinence Questionnaire-Short Form.

**Resultados:**

Os participantes do grupo de intervenção apresentaram melhores resultados quanto à redução do impacto da incontinência urinária na qualidade de vida (p ≤ 0,001), com destaque para limitações nas atividades diárias, percepção geral de saúde, limitações físicas e sociais, emoções, sono e humor.

**Conclusão:**

O programa cognitivo-comportamental foi eficaz na redução do impacto da incontinência urinária na qualidade de vida. Este estudo contribui para a prática clínica ao fornecer uma terapia eficaz, de baixo custo e de fácil aplicação. Registro Brasileiro de Ensaios Clínicos: RBR-3sstqg.

## RESUMEN

**Objetivo:**

Explorar los efectos de un programa cognitivo-conductual que aborda la incontinencia urinaria en la calidad de vida de los hombres que se han sometido a una prostatectomía radical.

**Método:**

Ensayo clínico controlado aleatorizado con pacientes sometidos a prostatectomía radical en una institución de tratamiento del cáncer en Brasil. Los 34 participantes fueron distribuidos aleatoriamente en dos grupos: 17 en el grupo de control que recibieron la atención estándar de la institución y 17 en el grupo de intervención que recibieron el programa cognitivo-conductual. La calidad de vida se evaluó utilizando el Cuestionario de Salud del Rey y el Cuestionario de Consulta Internacional sobre Incontinencia-Formulario Abreviado.

**Resultados:**

Los participantes del grupo de intervención mostraron mejores resultados en cuanto a la reducción del impacto de la incontinencia urinaria en la calidad de vida (p ≤ 0,001), con énfasis en las limitaciones en las actividades diarias, la percepción general de salud, las limitaciones físicas y sociales, las emociones y el sueño y el estado de ánimo.

**Conclusión:**

El programa cognitivo-conductual fue eficaz para reducir el impacto de la incontinencia urinaria en la calidad de vida. Este estudio contribuye a la práctica clínica al proporcionar una terapia efectiva, de bajo costo y de fácil aplicación. Registro Brasileño de Ensayos Clínicos: RBR-3sstqg.

## INTRODUCTION

Prostate cancer is the most common malignant disease in the male reproductive system^(1)^. The worldwide incidence of prostate cancer ranks second among malignant tumors in males, and fourth in relation to causes of death in both sexes^(2)^. There is a high incidence of prostate cancer in Brazil and, in recent years, with the aging of the population, changes in lifestyle, and the expansion of diagnostic tests, the incidence rate has presented a fast growth rate^(3)^.

Radical prostatectomy (RP) is the gold standard treatment for localized prostate cancer, but it is associated with complications such as urinary incontinence (UI) that results in impacts on quality of life (QoL)^(4)^. It is estimated that, within one year of surgery, 57% of men may experience UI^(5)^ and its occurrence is related to the clinical aspects of prostate cancer, patient age, intraoperative injuries, surgical methods, and surgeons’ experience^(6)^.

Post-radical prostatectomy UI (PRPUI) may be associated with urinary tract infections, perineal region dermatitis, and may also cause psychological distress, such as feelings of disability, uncertainty in life, stigma, reduced patient confidence in their treatment, changes in self-esteem and self-image, which can lead to pessimism, irritability, self-imposed social isolation, and, therefore, have detrimental effects on QoL^(7)^. Thus, PRPUI may hinder physical and mental rehabilitation^(8)^.

The active and early management of PRPUI improves postoperative recovery, helping to alleviate urinary complaints and resulting in the improvement of QoL of patients and their partners^(4)^. For PRPUI cases without nerve damage, the International Continence Society^(5)^ recommends behavioral measures such as changes in lifestyle habits and pelvic floor muscle training as the first choice of treatment. Level I and grade A evidence supports the effectiveness of behavioral measures for controlling PRPUI^(5)^. However, the challenge lies in patient adherence to behavioral measures to control UI (cognitive-behavioral approach to UI), which impacts the success rate of therapy^(9)^.

The cognitive therapy described by Bandura^(10)^ is a relevant approach for patient adherence to behavioral interventions used to control PRPUI, since it defines the relationship between behavioral interventions associated with cognitive strategies of social persuasion and positive feedback. A study that aimed to identify factors influencing adherence to pelvic floor muscle training in adult populations suggests that the cognitive behavioral approach can favor self-efficacy and improve men’s health-seeking behavior^(11)^. Thus, the discussing additional strategies, implemented by qualified professionals, may favor patient adherence and define the success rate of behavioral therapy. These strategies should integrate patients’ routine guidelines, monitoring the therapy process, and allowing persuasion and motivation mechanisms.

A quasi-experimental study using a sample with 71 men evaluated the effect of a cognitive behavioral program to control lower urinary tract symptoms (LUTS) in men after they had undergone prostate cancer treatment and showed a significant improvement in urinary symptoms (p ≤ 0.005) measured using the International Prostate Symptom Scores (IPSS) and bladder diaries^(12)^. Another result of the research was based on a reduction in emotional distress and a consequent positive impact on QoL. The authors highlight the relevance of clinical trial-type studies and the importance of individual and group therapies in this specific population.

Despite being considered a promising intervention in the care of men with PRPUI, there are few studies available in the literature on the effectiveness of the behavioral approach^(13)^, which hinders defining the real effectiveness of purely behavioral interventions. The authors focus on physical therapies using biofeedback and electrostimulation^(14)^, which are expensive and difficult to afford in healthcare services, or on a cognitive-behavioral approach to UI that have been directed to women^(15)^ and have little relevance and applicability for men with PRPUI.

Considering the advances in studies on the control mechanisms of male urinary continence based on transperitoneal ultrasound, it is known that the use of female UI rehabilitation protocols for men with PRPUI is not suitable^(16)^. Thus, the present study aimed to explore the effects of a cognitive-behavioral program on the impact of UI on the QoL of men who have undergone radical prostatectomy.

## METHOD

### Study Design

This is a single-blind, randomized clinical trial, following the guidelines of the Consort (2010)^(17)^. The study was carried out from November 2019 to December 2020 in an outpatient unit of a hospital institution specialized in oncology in the Midwest region of Brazil.

### Participants

The population consisted of men with PRPUI after the removal of the indwelling bladder catheter. The inclusion criteria were: men aged over 18 with mild, moderate, or severe UI who have been evaluated using the pad test^(18)^; having preserved locomotor, visual, auditory, and cognitive abilities (Mini Mental State Examination)^(19)^; having available telephone contact to receive the intervention, and availability for biweekly follow-ups. Patients with other diseases (diabetes mellitus, neurological injuries) or men who have used an indwelling urinary catheter for more than 21 days were excluded.

The pad test is a clinical instrument recommended by the International Continence Society, which consists of placing a penile pad near the external urethral meatus to quantify urine loss by comparing the weight of the pad before and after one hour. During this period, the patient undergoes a protocol of water intake and daily life activities^(18)^. Based on the difference in the weight of the initial and final pad, urinary losses are classified as: insignificant or continent loss (when the pad final weight is up to one gram (g); mild loss (1.1 to 9.9 g); moderate loss (10.0 to 49.9 g); severe loss (over 50.0 g).

The participants identification was carried out through a surgical and outpatient schedule of the institution. Patients were approached when they returned for the removal of their indwelling urinary catheter and were introduced to the project objectives. Upon demonstrating interest, patients were invited to return in 15 to 20 days to be informed of the study objectives and to sign an informed consent form.

### Sample Size

The sample calculation was based on the population of the study, consisting of 117 men undergoing RP. The sample size calculation was performed using the GPower^®^ software, version 3.1. Based on a significance level of 1% and a power of 95%, the calculation was based on the primary endpoint impact of UI on QoL (Incontinence Questionnaire – Short Form – ICIQ-SF) of the participants in the intervention group (IG) compared to the control group (CG) after three months of treatment^(20)^. The mean and standard deviation values of the CG and IG were 14.27 (± 3.25) and 9.03 (± 3.55), respectively. The calculation estimated a minimum value of 15 individuals in each group.

### Randomization and Blinding

The randomization was carried out by a researcher who was external to the study, in a 1:1 ratio, in two blocks of 17 people. Thus, two lists with 17 random numbers corresponding to the letter “I” (intervention) or “C” (control) were generated via the website (http://www.randomization.com/). Subsequently, from these lists, opaque mini envelopes were made for the generated codes (number+letter), which defined the group the research subject had been allocated to. Each study participant was assigned a unique study number in a sequential format (10 I or 11 C), ensuring that the randomization process was transparent and unbiased. Thus, after the data collection instruments had been applied and immediately before the intervention, the mini envelope was opened by the professional who applied the intervention to find out which group the participant would be allocated to. Blinding was strictly maintained until the database was finalized. A blinded evaluator conducted the evaluations after the program, while a non-blinded researcher, who was knowledgeable about the cognitive behavioral program, conducted separate visits during the program to provide the guidelines.

### Intervention Program

Participants in the CG were instructed to follow the routine guidelines provided by the service, which included care only for the surgical incision and no guidelines related to voiding control. The IG participants received interventions contained in a cognitive-behavioral program to control PRPUI.

The cognitive-behavioral program was developed based on the Social Cognitive Theory^(10)^ and was structured around two approaches, namely: face-to-face follow-up and remote follow-up. These approaches used strategies such as printed educational manuals, a vicarious experience video, a telephone follow-up script, and text messages.

Other strategies proposed by the Social Cognitive Theory^(10)^ were also used to encourage adherence to the interventions contained in the cognitive-behavioral program, such as social persuasion, positive feedback, and vicarious reinforcement (vicarious experience).

For the face-to-face approach, outpatient appointments were scheduled at four different times, namely: T0 (first moment – 15 to 20 days after indwelling urinary catheter removal); T1 (second moment – 30 days after T0); T2 (third moment – 60 days after T0), and T3 (fourth moment – 90 days after T0). These follow-ups lasted an average of 60 minutes (Chart 1).

**Chart 1:** Description of the intervention “Cognitive behavioral program to control Post-radical prostatectomy Urinary incontinence” – Goiânia, GO, Brazil, 2024.

**Cognitive-behavioral program to control PRPUI**		**Approaches**	
		**Face-to-face monitoring**	**Remote monitoring**
	**Strategies used**	– **Printed *manual***: *“Guidelines manual on post-radical prostatectomy urinary incontinence”* ^(21)^ with 39 pages which include texts and illustrations referring to guidelines on: anatomical structures involved in the control of Post-radical prostatectomy Urinary incontinence; habits interfering with micturition management (tobacco and caffeine cessation, adequate fluid intake; management of irritating foods, or foods that cause constipation); information on bladder re-education and training; a 3-month pelvic floor muscle training program: six stages that differed on the frequency (10 or 15 contractions) and the type of contractions (maximum and submaximal, sustained or unsustained), as well as body position during the exercises (lying down, sitting, standing, and walking). – **Video of vicarious experience**: a six-minute recording of a 73-year-old man who had Post-radical prostatectomy Urinary incontinence and underwent an urinary incontinence rehabilitation protocol similar to the program proposed in this study. He talks about his successful treatment to regain continence and improve his Quality of life.	– **Telephone follow-up script:** a guiding tool for the researcher to communicate with the participant over the phone. Structured by the authors, it consists of 17 questions about adherence to the guidelines received in person and contained in the printed manual (perception of self-efficacy, behavioral changes and pelvic floor muscle training). – **Text messages:** written communications not exceeding 100 words, sent via mobile device. The texts referred to reminders to perform pelvic floor muscle training, incentives and motivations to increase adherence to the behavioral habits described in the manual.
	**Description**	– Face-to-face meetings were conducted by the researcher in a private room at the healthcare service to guide and supervise the interventions proposed in the cognitive behavioral program; – Verbal and printed guidance was provided based on the printed manual; – During the service, the researcher assessed how the participant performed the pelvic floor muscle training exercises and advised improvements/stage advances as described in the printed manual.	– Weekly telephone contact was made according to the script and the participant’s demands. – Text messages were sent via the mobile phone of the researcher in charge.
	**Moment**	– Four monthly face-to-face moments: T0 (first moment): 15 to 20 days after the removal of the indwelling bladder catheter; T1 (second moment): 30 days after T0; T2 (third moment): 60 days after T0; T3 (fourth moment): 90 days after T0. – The video was shown in T0 (first moment), at the end of the verbal and printed guidelines for the meeting.	– Weekly telephone contacts and daily text messages during the three months of follow-up.
	**SCT Principle**	– **Positive *feedback* and social persuasion:** promoting a sense of self-confidence; – **Vicarious experience:** contributing factor to increased self-efficacy and adherence.	– **Positive *feedback* and social persuasion:** promoting a sense of self-confidence.

### Outcomes

The sociodemographic and clinical questionnaire evaluated sociodemographic, economic, clinical-surgical, and behavioral data. The variables investigated were: age, education, monthly income, occupation, marital status, body mass index, coffee/tea intake, alcohol consumption and smoking, regular physical activity, number of daily pads used and postoperative time.

The ICIQ-SF was used to measure UI primary endpoint impact on QoL, considered the primary outcome of the study. It consists of four questions referring to the frequency, severity, impact of UI, and self-diagnosis related to the causes or situations of UI experienced, respectively. The total score ranges from zero to 21, and the higher the value, the greater the impact on quality of life. In the present sample, the ICIQ-SF showed a Cronbach alpha value of 0.72^(22)^.

The King’s Health Questionnaire (KHQ) was also used to measure the impact of UI on QoL. The KHQ consists of 21 questions, subdivided into eight domains (general health perception, impact of UI, limitations on daily activities, physical limitations, social limitations, personal relationships, emotions, sleep and mood, and severity measurements), which were considered as secondary outcomes of the study. In addition to these domains, there is an independent scale that assesses the presence and intensity of urinary symptoms (Urinary Symptoms Scale). The responses to each of the items are Likert-type, graded in four response options (“0 – not at all, 1 – a little, 2 – moderately, 3 – a lot” or “0 – never, 1 – sometimes, 2 – frequently, 3 – all the time”), with the exception of the general perception of health domain with five answer options (“0 – very good, 1 – good, 2 – regular, 3 – bad, 4 – very bad”) and the personal relationships domain (“0 – not applicable, 1 – not at all, 2 – a little, 3 – moderately, and 4 – a lot”). The questionnaire is scored according to the domains, and there is no overall score. The scores range from 0 to 100 and, the higher the score, the greater the impact on QoL in the domain of interest^(23)^.

The outcomes measured by the ICIQ-SF and KHQ were assessed at baseline (T0) and at the different assessment times (T1, T2, and T3).

### Ethical Aspects

The study complied with Resolution 466/12 of the Brazilian National Health Council. It was approved by the Human Research Ethics Committee with number CAAE 80906217.3.3001.0031 and registered at the Brazilian Registry of Clinical Trials (RBR-3sstqg) (https://ensaiosclinicos.gov.br/). In addition, the Informed Consent Form was read and signed before data collection began.

### Statistical Analysis

For data analysis, the Statistical Package for the Social Sciences (SPSS) software for Windows^®^, version 23 was used. The Shapiro-Wilk test was performed to test whether the variables studied followed normal distribution. The nominal explanatory variables were described by frequency distribution and tables, while the quantitative variables, depending on normality, were described by the measurements of central tendency and dispersion: mean/standard deviation – normal distribution or median/percentiles – non-normal distribution. To evaluate the equivalence between groups regarding socio-demographic and clinical parameters in the pre-test, the Student’s t-test and the Mann-Whitney test were used. The Chi-squared or Fisher’s Exact test were used for categorical variables.

Regarding the comparison between IG and CG at different moments of the post-test and of each group over time, the outcomes at different time intervals were analyzed by the longitudinal model using the Generalized Estimating Equations (GEE) to evaluate the effect of group allocation, time, and the interaction between the effect of group and time (group*time). For significant effects at 5%, the comparison of the means was obtained using the post-hoc t-test protected by Bonferroni.

## RESULTS

### Participants

**Figure 1:**
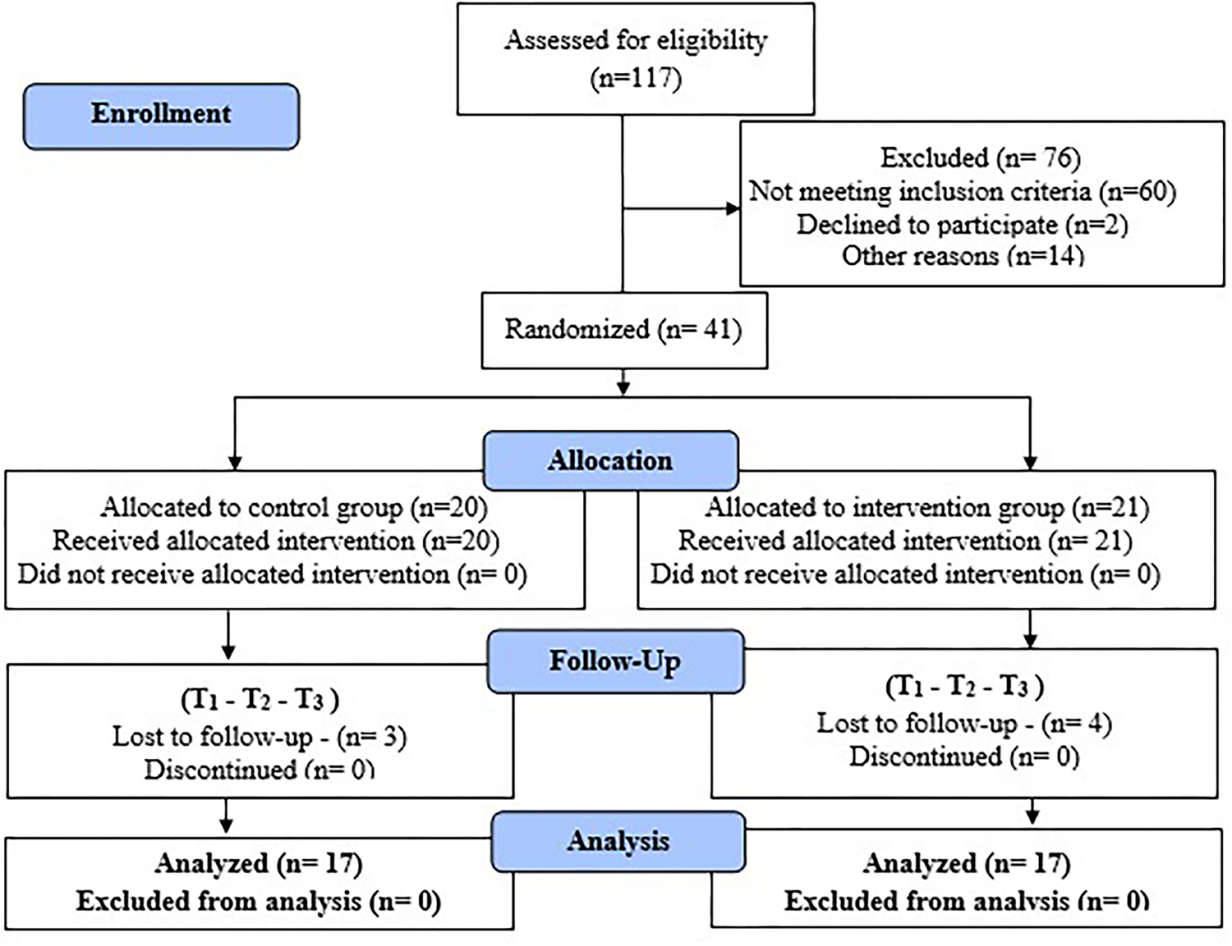
Flowchart of the participants included and analyzed in the study, in compliance with the Consolidated Standards of Reporting Trials – CONSORT. Goiânia, GO, Brazil.

Fifty-seven men undergoing RP were screened for eligibility, and 41 were eligible and randomized between CG and IG. A total of 21 men were allocated to the IG and 20 to the CG; four from the IG and three from the CG did not remain until the end of follow-up because of inability to return to face-to-face appointments due to the COVID-19 pandemic. Thus, 17 participants in the IG and 17 in the CG comprised the sample for outcome analysis. The participant recruitment flowchart is detailed in Figure 1.

The sociodemographic and clinical variables analyzed showed homogeneity between the groups, and consequently, the suitability of the randomization process (Table 1). No differences between the two groups on participants’ sociodemographic, clinical, and behavioral data were found at baseline (T0). The exception was the occupation variable (p < 0.011), in which the percentage of self-employed participants was higher in the CG (41.2%) than in the IG (5.9%), and the percentage of retired people in the IG (94.1%) exceeded that of the CG (35.3%).

**Table 1:** Sample characteristics regarding control group and intervention group (n = 34) – Goiânia, GO, Brazil, 2024.

Variables	Intervention group	Control group	p-value
**Age** ^ǂ^	66.7(6.0)	63.3(6.2)	0.117*
**Education** ^†^			
No education and <1 year	–	1(5.9)	0.474^#^
From 1 to 3 years	8(47.1)	5(35.3)	
From 4 to 7 years	8(47.1)	10(58.8)	
From 8 to 10 years	1(5.9)	–	
From 11 to 14 years	–	1(5.9)	
**Income (minimum wages)** ^†^			
Up to one	1(5.9)	1(5.9)	1.00^#^
From one to two times	10(58.8)	11(64.7)	
From two to four times	6(35.3)	5(14.7)	
**Occupation** ^†^			
Autonomous	1(5.9)	7(41.2)	**0.011** ^#^
Public servant	–	1(5.9)	
Private company employee	–	2(11.8)	
Unemployed	–	1(5.9)	
Retired	16(94.1)	6(35.3)	
**Marital status** ^†^			
With a partner	16(47.1)	16(47.1)	1.000^#^
Without a partner	1(5.9)	1(5.9)	
**BMI** ^µ^	23.9(4.6)	26.3(5.1)	0.163*
**Coffee/tea intake** ^†^			
No	3(17.6)	2(11.8)	1.000*
Yes	14(82.4)	15(88.2)	
**Alcoholic beverage consumption**			
No	13(38.2)	8(23.5)	0.078*
Yes	4(11.8)	9(26.5)	
**Smoker** ^†^			
No	14(82.4)	13(76.5)	0.671*
Yes	3(17.6)	4(23.5)	
**Regular physical activity** ^†^			
No	13(38.2)	12(35.3)	1.000*
Yes	4(11.8)	5(14.7)	
**Number of diapers used after IUC removal** ^ǂ^	3(2.0–4.5)	5(3.5–5.0)	0.786**
**Post-surgery time (days)** ^ǂ^	31(30–33)	31(29.5–33)	0.634**

*Student’s T-test;

**Mann-Whitney test;

^#^Fisher’s Exact Test;

^##^Chi-squared Test;

^µ^Mean;

^ǂ^Median(percentile25-percentile75);

^†^n(%).

Note: BMI: Body Mass Index; IUC: Indwelling Urinary Catheter.

Regarding the results of the impact of UI on the QoL measured by the ICIQ-SF and KHQ at baseline (T0) in the distinct assessment moments (T1, T2, and T3), for all domains there was a statistically significant difference between the groups at all assessment times (Table 2), with the sole exception of the personal relationships domain of the KHQ.

**Table 2:** Impact of the program on participants’ quality of life based on Generalized Estimating Equations (GEE), (n = 34) – Goiânia, GO, Brazil, 2024.

ICIQ-SF	T_0_ – m(sd)	T_1_ – m(sd)	T_2_ – m(sd)	T_3_ – m(sd)	T0–T1 – CI 95% p–value^£^	T0–T2 – CI 95% p-value^£^	T0–T3 – CI 95% p-value^£^
CG	17.6(0.7)	16.5(1.0)	15.5(1.2)	14.8(1.1)	–0.9; 3.0 / 0.966	–0.4; 4.5 / 0.178	0.8; 4.7 / **<0.001**
IG	16.3(0.8)	11.0(1.1)	6.5(0.7)	2.9(1.0)	1.8; 8.8 / **<0.001**	6.7; 12.9 / **<0.001**	9.1;17.2 / **<0.001**
CI 95%^†^	–3.4; 0.8	–8.7; –2.4	–11.9; –6.2	–15.0; –8.9			
p-value	0.246	**<0.001**	**<0.001**	**<0.001**			
**KHQ**							
**General health perception**							
CG	47.2(3.3)	48.2(2.3)	40.0(2.8)	41.1(3.1)	–6.5; 4.5 / 1.0	–0.9; 15.4 / 0.117	–1.96; 14.1 / 0.276
IG	43.5(3.4)	35.2(3.1)	28.3(2.9)	27.0(2.8)	–4.9; 21.4 / 0.600	1.5; 28.7 / **0.020**	3.1; 29.9 / **0.007**
CI 95%^†^	–13.1; 5.7	–20.6; –5.2	–19.7; –3.5	–22.4; –5.8			
p-value	0.440	**0.001**	**0.005**	**0.001**			
**Impact of UI**							
CG	88.8(3.7)	77.9(6.1)	73.5(7.0)	77.9(6.1)	–3.5; 25.3 / 0.278	–6.5; 37.1 / 0.384	–2.4; 24.2 / 0.187
IG	83.8(3.5)	57.3(5.4)	33.3(2.8)	32.7(4.2)	9.5; 43.3/ **<0.001**	38.5;62.4 /**<0.001**	34.7; 67.3 / **<0.001**
CI 95%^†^	–15.1; 5.0	–36.7; –4.4	–55.0; –25.3	–59.9; –30.4			
p-value	0.329	**0.012**	**<0.001**	**<0.001**			
**Limitations on daily activities**							
CG	59.5(4.8)	56.6(5.4)	47.7(4.0)	47.0(4.2)	–7.8; 13.7 / 1.000	–3.6; 27.1 / 0.262	–0.6; 25.6 / 0.073
IG	55.1(5.6)	34.5(3.0)	30.1(3.0)	26.4(1.4)	4.6; 36.5 / **0.004**	6.5; 43.3 / **0.002**	12.2; 45.0 / **<0.001**
CI 95%^†^	–18.9; 10.0	–34.2; –9.8	–27.6; –7.6	–29.3; –11.8			
p-value	0.551	**<0.001**	**0.001**	**<0.001**			
**Physical and social limitations**							
CG	50.7(3.8)	52.2(4.2)	50.0(4.4)	46.3(3.4)	–9.1; 6.2 / 1.000	–9.9; 11.4 / .000	–3.6; 12.4 / 0.901
IG	44.8(4.3)	31.6(1.8)	27.2(1.1)	26.8(1.1)	3.3; 23.1 / **0.002**	5.0; 30.2 / **<0.001**	5.3; 30.7 / **0.001**
CI 95%^†^	–17.1; 5.4	–29.6; –11.5	–31.8; –13.7	–26.6; –12.3			
p-value	0.308	**<0.001**	**<0.001**	**<0.001**			
**Personal relationships**							
CG	36.0(2.8)	41.1(4.3)	41.1(4.3)	42.3(3.1)	–17.0; 6.8 / 1.000	–14.8; 3.9 / 0.831	–15.4; 2.9 / 0.430
IG	34.1(1.4)	35.2(0.9)	33.3(1.2)	35.6(1.7)	–6.2; 3.9 / 1.000	–4.4; 6.0 / 1.000	–6.5; 3.4 / 1.000
CI 95%^†^	–8.1; 4.2	–14.5; 2.8	–16.6; 0.9	–13.7; 0.3			
p-value	0.538	0.185	0.080	0.063			
**Emotions**							
CG	49.9(3.7)	50.9(4.0)	50.9(5.2)	51.9(3.5)	–11.6; 9.6 / 1.000	–15.8; 13.8 / 1.000	–15.9; 12.0 / 1.000
IG	46.5(5.5)	30.3(2.0)	25.0(0.0)	25.0(0.0)	4.2; 28.0 / **0.002**	6.8; 36.3 / **<0.001**	6.8; 36.3 / **<0.001**
CI 95%^†^	–16.6; 9.8	–29.5; –11.5	–36.2; –15.7	–33.9; –19.9			
p-value	0.612	**<0.001**	**<0.001**	**<0.001**			
**Sleep and mood**							
CG	53.9(6.2)	48.5(5.1)	45.5(4.6)	48.5(4.3)	–2.8; 13.6 / 0.511	–5.9; 22.6 / 0.750	–5.9; 16.6 / 1.000
IG	42.6(4.8)	29.4(1.4)	25.7(0.7)	25.0(0.0)	1.7; 24.7 / **0.014**	3.4; 30.3 / **0.005**	4.7; 30.5 / **0.002**
CI 95%^†^	–26.7; 4.2	–29.6; –8.5	–29.0; –10.6	–32.1; –14.9			
p-value	0.155	**<0.001**	**<0.001**	**<0.001**			
**Severity measurements**							
CG	73.6(3.8)	66.4(4.1)	60.5(4.5)	65.2(3.8)	–2.7; 17.0 / 0.335	0.8; 25.2 / **0.029**	–8.2; 24.9 / 1.000
IG	72.6(2.5)	62.9(2.3)	42.6(2.7)	37.6(3.6)	–0.08; 19.4/0.053	19.4; 40.5 / **<0.001**	21.4; 48.5 / **<0.001**
CI 95%^†^	–10.0; 8.0	–12.8; 5.8	–28.4; –7.4	–37.9; –17.3			
p-value	0.830	0.458	**<0.001**	**<0.001**			

^£^Confidence interval for the 95% difference in the means; *p < 0.05 according to Bonferroni’s post hoc test.

Note: CG: Control group; IG: Intervention group; ICIQ-SF: Incontinence Questionnaire – Short Form; KHQ: King’s Health Questionnaire; m: Mean; sd: Standard deviation.

For the “severity measurements” domain, the effect could be proven at the first month of follow-up (p < 0.001), while for the “general health perception” domain, the effect could be observed from the second month of follow-up onward (p < 0.001). Thus, participants from the IG showed better results regarding the reduction of the impact of UI on QoL (p < 0.001), with emphasis on daily activity limitations, general perception of health, physical and social limitations, emotions, sleep, and mood (Table 2).

## DISCUSSION

The aim of this study was to evaluate the effectiveness of a cognitive behavioral program based on CBT in reducing the impact of UI on the QoL of men with PRPUI. The results show that there was a statistically significant reduction in the impact of UI on QoL in the IG, as assessed by the ICIQ-SF instrument, with a reduction of 13.4 points in the IG and only 2.8 points in the CG. There was also a reduction in the impact of UI on QoL using the KHQ domains: limitations on daily activities, general perception of health, physical and social limitations, emotions, and sleep and mood.

In this sense, the findings highlight the clinical success of the cognitive behavioral program based on social persuasion, positive feedback, and vicarious experience. It is therefore a promising and viable intervention capable of promoting greater adherence to the therapeutic process^(9,24)^. A quasi-experimental study that tested the effect of a cognitive-behavioral program to control urinary symptoms after prostate cancer treatment^(12)^ found similar results to those of the present study, with a reduction in the impact of UI on QoL. The intervention consisted of individual and group meetings through a cognitive component (teaching skills to improve coping, providing oral and written information, and emotional support), and a behavioral component (teaching pelvic floor muscle training and lifestyle changes)^(12)^.

Another relevant finding in this study was the satisfactory effect of combining written (booklet) and oral information in the implementation of the cognitive behavioral program. Brazilian researchers evaluated the use of a booklet on general postoperative care for prostatectomized men, and found that promoting patient education is key to improving self-care, minimizing anxiety, and thus favoring QoL^(24)^. Therefore, it is believed that men who only receive oral instructions on behavioral measures may find it more difficult to understand the treatment and this may have an impact on adherence and achieving the expected results.

Another strategy that has been widely developed and used in health education is the use of mobile health technologies. However, Chinese researchers^(25)^ presented an intervention protocol for UI self-management based on Social Cognitive Theory and structured it for mobile health technologies, explaining that making information available through digital tools does not exclude the need and importance of professional support through telephone contact and face-to-face meetings to clarify doubts, set goals, and increase the patient’s self-confidence.

The Social Cognitive Theory, often used in the development of interventions designed to modify behavior and increase self-efficacy, infers that an individual’s behavior is the result of the continuous interaction between the individual’s cognition, their environment, and behavioral factors. Personal experiences of success, vicarious experiences, social persuasion, and physiological states (reactions of the organism) are four main sources for increasing self-efficacy^(10)^. The ability to self-manage can also have a significant effect on self-efficacy, as it directly contributes to the acquisition of skills, which impacts on good performance and problem-solving abilities^(26)^.

It should be noted that vicarious experience is rarely used as a strategy to increase self-efficacy and there is a lack of studies exploring its effect on behavioral changes^(27)^. In the clinical context of prostatectomized men, the impacts of surgery on sexual function make this experience exchange among men more challenging, given the embarrassment and social stigmas associated with this health condition^(24)^. Although studies involving interventions with vicarious experience and behavioral measures to control UI are still in their infancy, it was possible to find a study with satisfactory results for increasing self-efficacy and decreasing the levels of depression in prostatectomized men based on a peer support intervention^(28)^.

The literature indicates^(29)^ that lower urinary tract symptoms, including UI, show clinical improvement within 12 months of RP; however, the impact on QoL during this period can be devastating. The involuntary loss of urine threatens the individual’s physical, social, economic and emotional performance, as well as causing a feeling of loss of masculinity, directly impacting on the affective lives of men undergoing RP. In this sense, support strategies to promote self-management in the treatment of UI are vital^(29)^. In addition, nursing care, which includes understanding the patient’s needs, education, and psychosocial support, continues to be an essential characteristic, aimed at improving patients’ quality of life^(25)^.

The results of the present study should be interpreted with caution regarding their generalization. The composition of the intervention and control groups was markedly different in terms of the number of retired participants (94.1% and 35.3%, respectively). Considering that the reduction of work activities is related to the improvement of health quality and well-being^(30)^, the effectiveness of the cognitive-behavioral program may have been influenced by the extended time availability that the retired participants in the intervention group had for exercising and changing their lifestyle habits. Another factor that requires caution for the external validity of the results is that this study included patients from an institution in a specific region of Brazil, which limits the possibility of generalizing the results for other cultural and social contexts.

Despite the aforementioned issues, the improvements in the instruments scores used suggest positive and significant impacts on clinical outcomes and on the enhancement of the perception of QoL. Thus, further future studies with the same participant profile are recommended to expand evidence.

## CONCLUSION

This study showed the positive effects of a cognitive-behavioral program on men’s QoL who have undergone RP. Participants who received the intervention showed a reduced impact of UI on their QoL, specifically improved general health perception, decreased physical, social, and daily activity limitations, improved emotions, sleep, and mood, and reduced self-perceived severity of PRPUI.

The intervention provided effective, low-cost, and easy-to-apply therapy. It is expected that the results presented will stimulate the implementation of this therapeutic program in other clinical practice scenarios for professionals in related areas. We believe that by incorporating these insights into clinical practice and public health strategies it will be possible to improve the health and QoL of men with post-radical prostatectomy UI.
